# 3-(Prop-2-yn-1-yl­oxy)phthalo­nitrile

**DOI:** 10.1107/S1600536813015663

**Published:** 2013-06-12

**Authors:** Chin Yee Jan, Norzianah Binti Haji Shamsudin, Ai Ling Tan, David J. Young, Edward R. T. Tiekink

**Affiliations:** a Faculty of Science, Universiti Brunei Darussalam, Jalan Tungku Link BE 1410, Negara Brunei Darussalam; b Department of Chemistry, University of Malaya, 50603 Kuala Lumpur, Malaysia

## Abstract

In the title compound, C_11_H_6_N_2_O {systematic name: 3-(prop-2-yn-1-yl­oxy)benzene-1,2-dicarbo­nitrile}, the 14 non-H atoms are approximately coplanar (r.m.s. deviation = 0.051 Å) with the terminal ethyne group being *syn* with the adjacent cyano residue. In the crystal, centrosymmetric dimers are connected by pairs of C—H⋯N inter­actions and these are linked into a supra­molecular tape parallel to (1-30) *via* C—H⋯N inter­actions involving the same N atom as acceptor.

## Related literature

For background to functionalized phthalocyanines, see: Chin *et al.* (2012 ▶). For background to the synthesis of precursor nitriles, see: Wu *et al.* (1998 ▶); Seven *et al.* (2009 ▶).
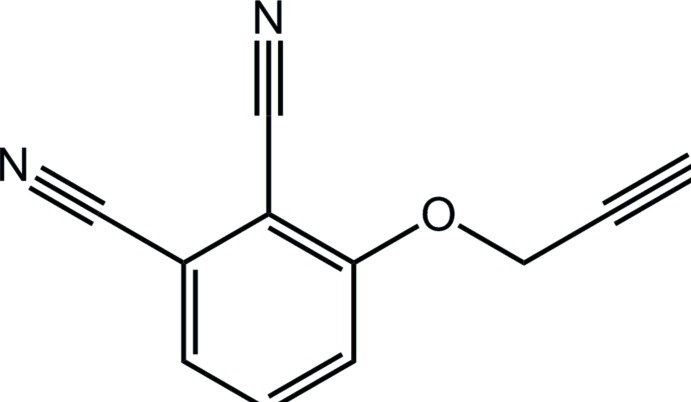


## Experimental

### 

#### Crystal data

C_11_H_6_N_2_O
*M* *_r_* = 182.18
Monoclinic, 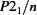

*a* = 4.014 (4) Å
*b* = 6.833 (7) Å
*c* = 33.85 (3) Å
β = 90.77 (2)°
*V* = 928.1 (16) Å^3^
*Z* = 4
Mo *K*α radiation
μ = 0.09 mm^−1^
*T* = 153 K
0.30 × 0.16 × 0.08 mm

#### Data collection

Bruker APEXII diffractometer
Absorption correction: multi-scan (*SADABS*; Sheldrick, 2003 ▶) *T*_min_ = 0.974, *T*_max_ = 0.993
6610 measured reflections
1751 independent reflections
1310 reflections with *I* > 2σ(*I*)
*R*_int_ = 0.071

#### Refinement

*R*[*F* ^2^ > 2σ(*F* ^2^)] = 0.086
*wR*(*F* ^2^) = 0.250
*S* = 1.13
1751 reflections
127 parameters
H-atom parameters constrained
Δρ_max_ = 0.28 e Å^−3^
Δρ_min_ = −0.31 e Å^−3^

### 

Data collection: *APEX2* (Bruker, 2011 ▶); cell refinement: *SAINT* (Bruker, 2011 ▶); data reduction: *SAINT*; program(s) used to solve structure: *SHELXS97* (Sheldrick, 2008 ▶); program(s) used to refine structure: *SHELXL97* (Sheldrick, 2008 ▶); molecular graphics: *ORTEP-3 for Windows* (Farrugia, 2012 ▶) and *DIAMOND* (Brandenburg, 2006 ▶); software used to prepare material for publication: *publCIF* (Westrip, 2010 ▶).

## Supplementary Material

Crystal structure: contains datablock(s) global, I. DOI: 10.1107/S1600536813015663/hg5321sup1.cif

Structure factors: contains datablock(s) I. DOI: 10.1107/S1600536813015663/hg5321Isup2.hkl

Supplementary material file. DOI: 10.1107/S1600536813015663/hg5321Isup3.cml

Additional supplementary materials:  crystallographic information; 3D view; checkCIF report

## Figures and Tables

**Table 1:** Hydrogen-bond geometry (Å, °)

*D*—H⋯*A*	*D*—H	H⋯*A*	*D*⋯*A*	*D*—H⋯*A*
C6—H6⋯N1^i^	0.95	2.47	3.335 (6)	151
C9—H9⋯N1^ii^	0.95	2.51	3.402 (6)	156

Symmetry codes: (i) 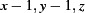; (ii) 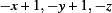.

**Fig. 1.**
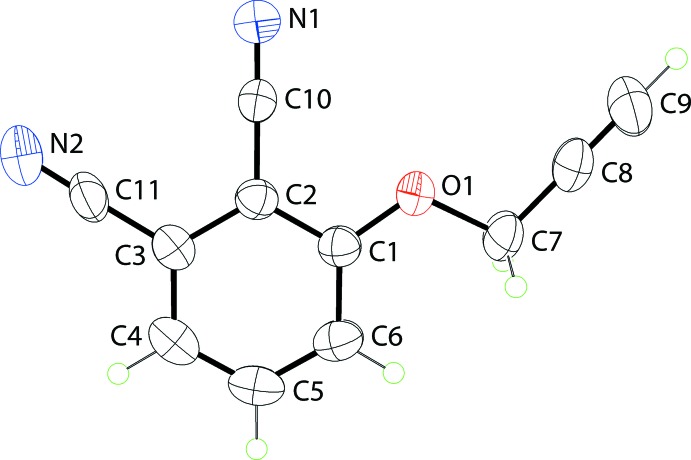
The molecular structure of (I) showing the atom-labelling scheme and displacement ellipsoids at the 50% probability level.

**Fig. 2.**
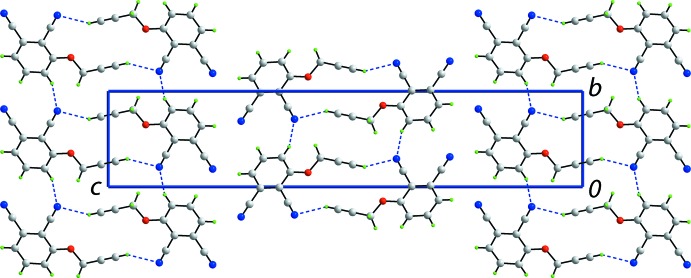
A view of the unit-cell contents of (I) in projection down the a axis. The C—H···N hydrogen bonds are shown as blue dashed lines.

## supplementary crystallographic information

### Comment

As part of our on-going study of functional phthalocyanines, we have previously reported the synthesis and structure (Chin *et al.*, 2012) of precursor 4-(prop-2-ylnyloxy)phthalonitrile obtained by the S_N_Ar reaction of propagyl alcohol and 4-nitrophthalonitrile, facilitated by potassium carbonate in DMF following literature precedents (Wu *et al.*, 1998; Seven *et al.*, 2009). Despite increased steric hindrance, this method is also suitable for preparing the title compound, (I).

In (I), Fig. 1, the 14 non-hydrogen atoms lie in a plane with the r.m.s. deviation of the fitted atoms being 0.051 Å; the maximum deviations from the least-squares plane are found for the C9 [0.102 (5) Å], and N1 and C7 [each -0.073 (4) Å] atoms. The ethyne group is *syn* to the adjacent cyano group. In the crystal structure, centrosymmetrically pairs are connected into dimeric aggregates *via* C—H···N interactions and these are in turn linked into a supramolecular tape, parallel to (1 - 3 0), *via* C—H···N interactions with translationally related dimeric aggregates, Fig. 2 and Table 1. As the N1 atom participates in both C—H···N interactions, it is bifurcated. Chains stack along the *a* axis with separations of 4.014 (4) Å between the benzene rings, corresponding to the length of the *a* axis, and with no significant intermolecular interactions between them.

### Experimental

The title compound was prepared by modification of literature procedures (Wu *et al.*, 1998; Seven *et al.*, 2009). Under a nitrogen atmosphere, anhydrous potassium carbonate (1.60 g, 11.6 mmol) was added in three portions at 1 h intervals to a solution of propargyl alcohol (2.16 ml, 37.4 mmol) and 3-nitrophthalonitrile (1.01 g, 5.83 mmol) in dry *N*,*N*-dimethylformamide (10 ml). After 96 h, the crude reaction mixture was poured into water (200 ml) and stirred rapidly. The brown precipitate was collected by vacuum filtration, washed with water and dried to provide 0.91 g of material that was purified by silica gel column chromatography using CH_2_Cl_2_ and recrystallized from CH_2_Cl_2_/hexane to yield 0.68 g (63.8%). *M*. pt: 427–429 K. IR (KBr) ν/cm^-1^: 3296, 3095, 2230, 2138, 1586, 1474, 1376, 1297. ^1^H NMR 400 MHz (CDCl_3_) δ: 7.68 (1*H*, m), 7.42 (2*H*, m), 4.91 (2*H*, *d*, J = 2.4 Hz), 2.62 (1*H*, *t*, J = 2.4 Hz).

### Refinement

Carbon-bound H-atoms were placed in calculated positions [C—H = 0.95–0.99 Å, *U*_iso_(H) = 1.2*U*_eq_(C)] and were included in the refinement in the riding model approximation.

### Crystal data

**Table:**

C_11_H_6_N_2_O	*F*(000) = 376
*M**_r_* = 182.18	*D*_x_ = 1.304 Mg m^−^^3^
Monoclinic, *P*2_1_/*n*	Mo *K*α radiation, λ = 0.71073 Å
Hall symbol: -P 2yn	Cell parameters from 2684 reflections
*a* = 4.014 (4) Å	θ = 2.4–28.4°
*b* = 6.833 (7) Å	µ = 0.09 mm^−^^1^
*c* = 33.85 (3) Å	*T* = 153 K
β = 90.77 (2)°	Prism, colourless
*V* = 928.1 (16) Å^3^	0.30 × 0.16 × 0.08 mm
*Z* = 4	

### Data collection

**Table:**

Bruker APEXII diffractometer	1751 independent reflections
Radiation source: fine-focus sealed tube	1310 reflections with *I* > 2σ(*I*)
Graphite monochromator	*R*_int_ = 0.071
φ and ω scans	θ_max_ = 25.7°, θ_min_ = 1.2°
Absorption correction: multi-scan (*SADABS*; Sheldrick, 2003)	*h* = −4→4
*T*_min_ = 0.974, *T*_max_ = 0.993	*k* = −8→8
6610 measured reflections	*l* = −40→41

### Refinement

**Table:**

Refinement on *F*^2^	Primary atom site location: structure-invariant direct methods
Least-squares matrix: full	Secondary atom site location: difference Fourier map
*R*[*F*^2^ > 2σ(*F*^2^)] = 0.086	Hydrogen site location: inferred from neighbouring sites
*wR*(*F*^2^) = 0.250	H-atom parameters constrained
*S* = 1.13	*w* = 1/[σ^2^(*F*_o_^2^) + (0.1092*P*)^2^ + 0.8456*P*] where *P* = (*F*_o_^2^ + 2*F*_c_^2^)/3
1751 reflections	(Δ/σ)_max_ < 0.001
127 parameters	Δρ_max_ = 0.28 e Å^−^^3^
0 restraints	Δρ_min_ = −0.31 e Å^−^^3^

### Special details

**Table:**

Geometry. All s.u.'s (except the s.u. in the dihedral angle between two l.s. planes) are estimated using the full covariance matrix. The cell s.u.'s are taken into account individually in the estimation of s.u.'s in distances, angles and torsion angles; correlations between s.u.'s in cell parameters are only used when they are defined by crystal symmetry. An approximate (isotropic) treatment of cell s.u.'s is used for estimating s.u.'s involving l.s. planes.
Refinement. Refinement of *F*^2^ against ALL reflections. The weighted *R*-factor *wR* and goodness of fit *S* are based on *F*^2^, conventional *R*-factors *R* are based on *F*, with *F* set to zero for negative *F*^2^. The threshold expression of *F*^2^ > 2σ(*F*^2^) is used only for calculating *R*-factors(gt) *etc*. and is not relevant to the choice of reflections for refinement. *R*-factors based on *F*^2^ are statistically about twice as large as those based on *F*, and *R*- factors based on ALL data will be even larger.

### Fractional atomic coordinates and isotropic or equivalent isotropic displacement parameters (Å^2^)

**Table:**

	*x*	*y*	*z*	*U*_iso_*/*U*_eq_	
O1	0.1024 (7)	0.3511 (3)	0.07894 (6)	0.0506 (7)	
N1	0.4013 (9)	0.7927 (4)	0.10745 (9)	0.0563 (9)	
N2	0.1893 (10)	0.8135 (5)	0.21998 (10)	0.0680 (11)	
C1	0.0004 (8)	0.3448 (5)	0.11659 (9)	0.0392 (8)	
C2	0.0883 (8)	0.5067 (5)	0.13953 (9)	0.0385 (8)	
C3	0.0045 (8)	0.5137 (5)	0.17948 (9)	0.0412 (8)	
C4	−0.1691 (9)	0.3613 (6)	0.19646 (11)	0.0526 (10)	
H4	−0.2270	0.3655	0.2236	0.063*	
C5	−0.2565 (9)	0.2033 (6)	0.17330 (11)	0.0525 (10)	
H5	−0.3765	0.0984	0.1848	0.063*	
C6	−0.1754 (9)	0.1927 (5)	0.13400 (11)	0.0486 (9)	
H6	−0.2392	0.0818	0.1188	0.058*	
C7	0.0045 (10)	0.1952 (6)	0.05273 (10)	0.0515 (10)	
H7A	−0.2412	0.1905	0.0499	0.062*	
H7B	0.0831	0.0678	0.0632	0.062*	
C8	0.1545 (9)	0.2349 (5)	0.01494 (10)	0.0498 (9)	
C9	0.2816 (12)	0.2662 (7)	−0.01551 (12)	0.0694 (13)	
H9	0.3844	0.2914	−0.0401	0.083*	
C10	0.2645 (8)	0.6647 (5)	0.12159 (9)	0.0404 (8)	
C11	0.1051 (9)	0.6805 (6)	0.20227 (10)	0.0495 (9)	

### Atomic displacement parameters (Å^2^)

**Table:**

	*U*^11^	*U*^22^	*U*^33^	*U*^12^	*U*^13^	*U*^23^
O1	0.0697 (17)	0.0452 (14)	0.0369 (13)	−0.0151 (12)	0.0050 (11)	−0.0086 (10)
N1	0.074 (2)	0.0469 (18)	0.0487 (18)	−0.0159 (16)	0.0095 (16)	−0.0035 (14)
N2	0.088 (3)	0.073 (2)	0.0424 (18)	−0.003 (2)	0.0059 (17)	−0.0152 (17)
C1	0.0385 (18)	0.0380 (17)	0.0410 (17)	−0.0019 (13)	−0.0001 (13)	0.0016 (13)
C2	0.0385 (17)	0.0362 (17)	0.0408 (17)	0.0010 (13)	0.0019 (13)	0.0024 (13)
C3	0.0407 (18)	0.0452 (19)	0.0376 (16)	0.0053 (15)	0.0020 (13)	0.0023 (14)
C4	0.053 (2)	0.063 (2)	0.0417 (18)	0.0042 (18)	0.0083 (16)	0.0119 (17)
C5	0.047 (2)	0.051 (2)	0.060 (2)	−0.0061 (17)	0.0055 (17)	0.0173 (18)
C6	0.048 (2)	0.0408 (19)	0.056 (2)	−0.0084 (15)	−0.0022 (16)	0.0037 (15)
C7	0.056 (2)	0.054 (2)	0.0438 (19)	−0.0129 (17)	−0.0041 (16)	−0.0145 (16)
C8	0.057 (2)	0.048 (2)	0.044 (2)	0.0008 (17)	−0.0083 (17)	−0.0126 (16)
C9	0.091 (3)	0.074 (3)	0.043 (2)	0.003 (2)	0.009 (2)	−0.009 (2)
C10	0.049 (2)	0.0364 (17)	0.0353 (17)	−0.0029 (15)	0.0020 (14)	−0.0048 (13)
C11	0.057 (2)	0.061 (2)	0.0313 (17)	0.0009 (18)	0.0076 (15)	−0.0044 (16)

### Geometric parameters (Å, º)

**Table:**

O1—C1	1.345 (4)	C4—C5	1.377 (6)
O1—C7	1.437 (4)	C4—H4	0.9500
N1—C10	1.141 (4)	C5—C6	1.376 (5)
N2—C11	1.137 (5)	C5—H5	0.9500
C1—C6	1.391 (5)	C6—H6	0.9500
C1—C2	1.394 (5)	C7—C8	1.447 (5)
C2—C3	1.399 (5)	C7—H7A	0.9900
C2—C10	1.431 (4)	C7—H7B	0.9900
C3—C4	1.382 (5)	C8—C9	1.176 (5)
C3—C11	1.431 (5)	C9—H9	0.9500
			
C1—O1—C7	118.5 (3)	C4—C5—H5	119.0
O1—C1—C6	126.0 (3)	C5—C6—C1	119.9 (3)
O1—C1—C2	115.1 (3)	C5—C6—H6	120.1
C6—C1—C2	118.9 (3)	C1—C6—H6	120.1
C1—C2—C3	120.2 (3)	O1—C7—C8	107.0 (3)
C1—C2—C10	119.0 (3)	O1—C7—H7A	110.3
C3—C2—C10	120.7 (3)	C8—C7—H7A	110.3
C4—C3—C2	120.3 (3)	O1—C7—H7B	110.3
C4—C3—C11	121.1 (3)	C8—C7—H7B	110.3
C2—C3—C11	118.6 (3)	H7A—C7—H7B	108.6
C5—C4—C3	118.7 (3)	C9—C8—C7	178.9 (4)
C5—C4—H4	120.7	C8—C9—H9	180.0
C3—C4—H4	120.7	N1—C10—C2	179.0 (4)
C6—C5—C4	122.0 (3)	N2—C11—C3	178.9 (4)
C6—C5—H5	119.0		
			
C7—O1—C1—C6	4.8 (5)	C11—C3—C4—C5	−179.3 (3)
C7—O1—C1—C2	−176.3 (3)	C3—C4—C5—C6	0.2 (6)
O1—C1—C2—C3	−178.1 (3)	C4—C5—C6—C1	0.0 (6)
C6—C1—C2—C3	0.9 (5)	O1—C1—C6—C5	178.2 (3)
O1—C1—C2—C10	2.0 (4)	C2—C1—C6—C5	−0.6 (5)
C6—C1—C2—C10	−179.0 (3)	C1—O1—C7—C8	−178.3 (3)
C1—C2—C3—C4	−0.7 (5)	O1—C7—C8—C9	74 (21)
C10—C2—C3—C4	179.2 (3)	C1—C2—C10—N1	123 (21)
C1—C2—C3—C11	178.7 (3)	C3—C2—C10—N1	−57 (21)
C10—C2—C3—C11	−1.4 (5)	C4—C3—C11—N2	153 (24)
C2—C3—C4—C5	0.1 (5)	C2—C3—C11—N2	−26 (25)

### Hydrogen-bond geometry (Å, º)

**Table:**

*D*—H···*A*	*D*—H	H···*A*	*D*···*A*	*D*—H···*A*
C6—H6···N1^i^	0.95	2.47	3.335 (6)	151
C9—H9···N1^ii^	0.95	2.51	3.402 (6)	156

Symmetry codes: (i) *x*−1, *y*−1, *z*; (ii) −*x*+1, −*y*+1, −*z*.
